# Association Between Bullying/Cyberbullying and Subjective Vitality in Children and Adolescents: The Mediating Role of Mental Toughness

**DOI:** 10.3390/pediatric17020027

**Published:** 2025-02-25

**Authors:** Pablo Ramírez-Espejo, Alba Rusillo-Magdaleno, Alberto Ruiz-Ariza, Manuel J. de la Torre-Cruz

**Affiliations:** 1 Musical, Plastic and Corporal Expression Didactics Department, Faculty of Humanities and Educational Sciences, University of Jaén, 23071 Jaén, Spain; pre00005@red.ujaen.es (P.R.-E.); arusillo@ujaen.es (A.R.-M.); arariza@ujaen.es (A.R.-A.); 2 Department of Psychology, Faculty of Humanities and Educational Sciences, University of Jaén, 23071 Jaén, Spain

**Keywords:** victimization, aggression, adolescent, mental toughness, subjective vitality, mediation analysis

## Abstract

**Background:** Previous research has examined to what extent the relationship between bullying/cyberbullying (victims and perpetrators) and psychological well-being is mediated by different cognitive–emotional variables. **Objectives:** The present study aimed to analyze whether mental toughness acted as a mediating variable in the relationship between bullying, suffered and perpetrated, and subjective vitality. **Methods:** Three hundred and twelve students in their final year of Primary Education and Compulsory Secondary Education, aged between 11 and 17 years, gave their responses to different self-report measures. **Results:** The results showed that perceived mental toughness significantly mediated the relationship between victimization and perpetration of aggressive acts through the use of electronic devices and levels of subjective vitality. A similar mediating effect was observed for occasions when the adolescent engaged in bullying acts during face-to-face interactions. **Conclusions:** It is concluded that high levels of mental toughness may act as a protective mechanism, reducing or mitigating the loss of subjective vitality resulting from being victimized in a virtual environment, as well as from perpetrating aggressive acts during face-to-face interactions and through the use of technological media.

## 1. Introduction

School bullying, whether experienced or perpetrated through face-to-face interactions (bullying) or through technological means (cyberbullying) is a recurring problem among students in educational institutions in developed countries [1,2,3]. Both forms of bullying are defined as manifestations of aggressive acts among peers, characterized by intentional and repeated behavior over time, involving a power imbalance between the perpetrator and the victim [4,5]. In the case of cyberbullying, harmful behaviors using technology as a conduit (text messages, comments in chats or on websites, spreading false rumors or testimonies) transcend spatial and temporal limitations [6]. Although physical harm is unlikely, the affective, relational or social harm caused can be more disruptive, as the victim may find it more difficult to resist such attacks due to the anonymity that technology provides to the aggressor [7].

The prevalence of these behaviors varies considerably among the various studies, differences that, among other reasons, have been attributed to the particular way of defining and assessing aggressive behavior towards peers [8]. Beyond this important appreciation, Modecki et al. [9] observed that the frequency of face-to-face aggressive acts, whether in the role of victim or perpetrator, was as high as 35%. This value is higher than the 16.5% reported by Anderson et al. [1] or the 6.1% reported by Machimbarrena et al. [10]. However, these percentages increase on occasions when aggressive acts are perpetrated or received through digital media. In this sense, Zych et al. [11], concluded that the average prevalence was 24.6% for cyber perpetration, as well as 26.6% for cybervictimization. More recently, Zhu et al. [12] analyzed the prevalence of this phenomenon in studies conducted in different countries and concluded that the rate of cybervictimization ranged from 14.6% to 52.2%, Spain being the country with the highest rate, while in the case of cyberaggression, the values ranged from 6.3% to 32%, China being the country with the highest figures. Participation in bullying or cyberbullying scenarios, whether in the role of victim, aggressor, or victim–aggressor, appears to be associated with the subjective well-being of children and adolescents [13,14]. Subjective well-being is an integral construct referring to a multiplicity of self-evaluations of life, in general, that brings together both cognitive and emotional elements [15] that are closely related to people’s mental and psychosocial health. In this regard, previous research shows that participation in bullying situations, both in face-to-face and virtual settings, is associated with reduced rates of adjustment, well-being, and mental health in children and adolescents, an association that continues even into early adulthood [16,17]. Specifically, the involvement or participation in aggressive acts is associated with psychological vulnerability, as manifested by increased depressive symptoms [18,19,20,21], anxiety [22,23], test and evaluation anxiety [24], feelings of isolation and loneliness and even the presence of suicidal ideation [25]. Also, Mark et al. [13] showed that the impact of bullying and cyberbullying on the mental health of children and adolescents is even more pernicious for boys and girls who simultaneously play the role of victim–perpetrator in unison.

Beyond the indicated relationships, Kowalski et al. [2] developed an explanatory model of aggressive behavior in which they considered the possibility that the relationship established between victimization and psychological well-being may be mediated by cognitive and emotional factors. In this sense, negative impressions such as feelings of isolation, rejection and guilt [26], or positive ones such as high self-esteem [27], higher perceived social support [28], and commitment to the school institution [29] or resilience [27,30] could either exacerbate or mitigate the psychological distress or maladjustment associated with the experience of school bullying. In line with this mediational approach, Li et al. [26] found that greater perceptions of cybervictimization increased feelings of psychological insecurity, which in turn, led to higher levels of depressive symptoms in boys and girls. Similarly, the results of the study conducted by Irwin et al. [31] revealed that the more guilt was attributed to feeling victimized, the greater the perceived psychological distress in terms of social anxiety and manifestation of externalizing behavioral problems. Meanwhile, Múzquiz et al. [32] reported that the relationship between bullying and adolescent emotional well-being was fully mediated by feelings of self-compassion. Greater acceptance and forgiveness for possible mistakes contributed to improved emotional well-being, thereby buffering the negative relationship between the bullying experience and well-being.

As stated by Li et al. [26], identifying mediating variables in the relationship between school bullying and emotional well-being is crucial for designing strategies to promote the psychological health of children and adolescents. One variable that has gained prominence in the last decade is resilience [30]. Resilience has been defined as the ability to achieve a positive adjustment to adverse stress-generating situations, a capacity that seems to be directly related to subjective well-being [33]. The results of several studies suggest that resilience plays a regulatory role in peer bullying and well-being. In this regard, Moore and Woodcock [33] observed that students who attributed a high level of mastery and belonging together with lower emotional reactivity, and qualities of resilience, showed fewer depressive symptoms after being victimized by their peers. In a similar vein, Gianesini and Brighi [34] found that the resilience of adolescents buffered the negative consequences of peer violence in cyberbullying situations. Specifically, more resilient victims appeared to possess greater regulatory skills, resources and competencies to cope with cyberbullying. More recently, Shemesh and Heiman [27] observed that the relationship between bullying victimization and adolescent subjective well-being was fully mediated by self-concept and resilience, a result that allowed them to conclude that a positive self-concept together with a high level of resilience would contribute to lessen the loss in subjective well-being associated with feeling victimized.

A different construct with a functional utility very similar to that of resilience, namely coping with threatening or adverse circumstances, is mental toughness [35,36,37]. According to Aslanyürek and Demircioglu [38], the difference between resilience and mental toughness lies in the fact that the former focuses on external resources and factors for coping with challenges while the latter appeals to personal or internal characteristics. Although its origin lies in the field of athletic preparation and competition [36], more recently mental toughness has been viewed as a domain of psychological functioning with significant implications for educational contexts as well as health and general well-being [39]. Mental toughness is defined as the innate or learned ability to cope effectively with stress and anxiety resulting from challenging situations [35]. High levels of mental toughness are associated with determination, confidence, and resilience in the face of adversity [40,41]. Studies conducted with adolescents and young adults in educational settings show that mental toughness is inversely related to perceived stress [40], the presence of depressive symptoms, emotional exhaustion, and physical fatigue [42], and directly related to psychological well-being [43,44] and academic performance [45].

As indicated in the preceding paragraphs, participation in bullying situations is linked to psychological well-being. In the words of Ryan and Frederick [15] the subjective feeling of vitality can act as a significant indicator of personal well-being. Subjective vitality refers to the conscious experience of feeling energetic, alive and enthusiastic [46]. Because of its alignment with positive phenomenological circumstances, subjective vitality has been linked to happiness, self-fulfillment, motivation and health, making it the quintessential indicator of eudaimonic well-being [47,48]. Qi et al. [49] indicate that subjective vitality is positively associated with both the meaning of life and psychological development in adolescents. Previous research has confirmed that subjective vitality is directly related to the use of appropriate coping strategies to deal with stress and adversity [50], self-esteem, life satisfaction, positive affect, intrinsic motivation [51] and the quality of peer relationships [52].

Subjective vitality has been used as an indicator of the positive affective component of subjective well-being. In this sense, subjective vitality should be higher to the extent that a person is free of conflicts. On the contrary, participation in conflict scenarios that threaten self-regulation, autonomy and self-fulfillment, such as bullying situations that generate nervousness, anxiety or pressure, could reduce the feeling of subjective vitality [15]. Also, as indicated in the preceding paragraphs, most studies have examined the role that various cognitive–emotional variables, such as resilience [27,33], play in the relationship between bullying and subjective well-being. However, to the best of our knowledge, none of the research conducted has considered the extent to which mental toughness, a construct that shares certain similarities with that of resilience, might mediate this relationship. Furthermore, research on the relationship between school bullying and psychological well-being has focused primarily on victimization and the negative aspects of well-being. Less attention has been paid to the dual dimension of victim–perpetrator roles and the variables that contribute to improving psychological well-being, such as subjective vitality.

Therefore, based on the aforementioned points, the present study aimed to examine the relationships between school bullying, mental toughness and subjective vitality in a sample of Spanish students enrolled in the last year of Primary Education and different years of Compulsory Secondary Education. Specifically, the extent to which mental toughness acts as a significant mediator in the relationship between bullying and cyberbullying (from both the victim and perpetrator perspectives) and perceived levels of subjective vitality were analyzed. It was hypothesized that greater mental toughness from the victim’s perspective (traditional bullying and cyberbullying) would help to moderate the likely negative relationship between perceived aggression and subjective vitality. Furthermore, an inverse relationship between perpetrated aggressive acts and subjective vitality was hypothesized. However, in the absence of prior empirical evidence, the potential mediating role of mental toughness in the latter case was not established. In any case, this mediation was expected to be higher for any of the forms of victimization with respect to perpetration.

## 2. Materials and Methods

### 2.1. Participants

A total of 312 students from the last year of Primary Education (sixth grade) and different years of Compulsory Secondary Education (from first to fourth year) aged between 11 and 17 years (mean age ± standard deviation: 13.32 ± 1.59; 52.8% girls) participated in this cross-sectional quantitative study. The students were drawn from six schools in the Autonomous Community of Andalusia, located in south of Spain. The selection of the schools was based on convenience sampling. Data collection and test administration took place between February and April 2023.

### 2.2. Procedure

Initially, the administrative teams of schools, as well as the parents, guardians or legal representatives of the students and the students themselves were informed in writing about the purpose of the study. For those schools that chose to collaborate, an informed consent form was provided for the primary caregivers to sign to authorize their child’s participation in the study. To maximize confidentiality and anonymity, participants’ personal details were replaced by alphanumeric codes. Students completed the measures of interest during school hours under the supervision of members of the research team and the school staff. A maximum of 45 min was allowed to complete the measures. The study was approved by the Bioethics Committee of the University of Jaen (Spain). The reference code for this approval was NOV.22/2.PRY; approved on 13 January 2023. The study design complied with the Spanish government guidelines on clinical research involving human subjects (Royal Decree 561/1993 on clinical trials), as well as the fundamental ethical principles established in the Declaration of Helsinki (2013, Brazil).

### 2.3. Instruments

#### 2.3.1. Sociodemographic Variables

First, the participating students provided information about their biological sex, age, and the academic year in which they were enrolled.

#### 2.3.2. Bullying

The Spanish adaptation of the European Bullying Intervention Project Questionnaire by Ortega Ruiz et al. [53], developed for students of Compulsory Secondary Education. was used to assess traditional school bullying (from both the victim and aggressor perspectives). The scale consists of a total of 14 items related to the perpetration (seven items) or experience (seven items) of actions such as hitting, stealing objects, threatening, insulting, spreading rumors or using offensive language towards peers, among others. The response format is a five-point Likert scale (from never = 0 to always = 4) indicating the frequency with which the described behaviors have been experienced or perpetrated in the past two months. A higher score on the scale corresponds to a greater degree of bullying experienced or perpetrated. The internal consistency indices obtained, measured using Cronbach’s alpha were 0.85 for victimization and 0.80 for aggression.

#### 2.3.3. Cyberbullying

Cyberbullying or bullying through electronic means was assessed with the Spanish version of the European Cyberbullying Intervention Project Questionnaire “ECIPQ” previously used in Compulsory Secondary Education students [54]. The questionnaire consists of 22 items relating to acts such as spreading rumors, social exclusion or identity theft, grouped into two dimensions: cybervictimization and cyberaggression, each of which consists of 11 items. Participant’s responses were recorded on a Likert-type scale measuring the frequency of exposure to or perpetration of the identified behaviors (from never = 0 to always = 4), over the past two months. A higher score corresponds to a greater degree of aggression experienced or exhibited through information and communication technologies. Ortega-Ruiz et al. [53] reported acceptable reliability indices (α = 0.80 cybervictimization and α = 0.88 cyberaggression) for each dimension. In the sample for this study, the internal consistency values were α = 0.78 and α = 0.78, respectively.

#### 2.3.4. Mental Toughness

Mental toughness was assessed using a reduced eight-item scale developed by Gucciardi et al. [55], based on the original proposal by Clough et al. [35]. This scale was validated in a sample of university students. Each item belongs to one of the identified dimensions of the mental toughness construct: self-efficacy (confidence in one’s ability to succeed in achievement contexts); attentional regulation (ability to focus on relevant information while ignoring other irrelevant information for a purpose); emotional regulation (awareness and ability to manage emotions in order to achieve a goal); success mindset (desire to achieve certain recognition and act motivated to achieve it); contextual knowledge (knowledge of the performance context and use of this knowledge to achieve objectives and goals); resilience (ability to execute the processes and skills required in response to daily challenges and pressures); optimism (tendency to expect positive events to occur in the near future) and; adversity coping (resilience in the face of problems). Participants were asked to respond to a seven-item Likert scale indicating the frequency (never = 1 to always = 7) with which they engage in or perform actions that reflect this mental toughness. A final score was calculated by averaging the responses to each item. A higher score was equated with a higher level of mental toughness. The internal consistency index for the study sample was α = 0.84.

#### 2.3.5. Subjective Vitality

Finally, the Spanish version adapted by Castillo et al. [46] of the Subjective Vitality Scale of [51] was used to assess the subjective experience of feeling vital and full of energy in a sample of Spanish adolescents (e.g., “I feel alive and vital”). The measure consists of seven items, where participants indicate their level of agreement on a Likert-type scale with seven response options (strongly disagree = 1 to strongly agree = 7) for each statement. Item number two (“I don’t feel very energetic”) had a negative impact on the reliability of the scale and was therefore excluded from the calculation of the final average score. A higher mean score corresponded to a higher perceived subjective vitality. The internal consistency index for this group of participants was α = 0.89.

### 2.4. Statistical Analysis

Descriptive statistics are presented as means and standard deviations. Reliability, understood as internal consistency, was calculated using Cronbach’s alpha test. The relationship between variables was analyzed using Pearson’s correlation coefficient. Finally, the PROCESS macro (Model 4) [56] for SPSS was used to conduct mediation analyses. Specifically, four mediation analyses were conducted with bullying and cyberbullying behaviors (from victim and aggressor perspectives) as the independent variables, mental toughness as the mediating variable and, subjective vitality as the dependent variable. Given that previous research has established that girls score lower than boys on variables associated with subjective well-being [13,57], as well as that psychological well-being decreases with increasing age during adolescence [58,59], sex and age were included as covariates in the mediation models. The SPSS macro allowed 10,000 bootstrap samples of the data with replacement to calculate the bias-corrected bootstrap point estimate for the size and significance of the indirect effect. All data were analyzed using SPSS, version 25.0 (IBM, Inc., SPSS, Chicago, IL, USA). Statistical significance was set at *p* < 0.05.

## 3. Results

### 3.1. Descriptive Statistics and Correlation Indexes

Table 1 shows mean values, standard deviations and Pearson correlation indexes among the different study variables. The analysis of the relationship between the variables showed that the age of the participant was inversely related to the frequency of feeling victimized by bullying, (r = −0.243, *p* < 0.01), mental toughness (r = −0.128, *p* < 0.05) and subjective vitality (r = −0.169, *p* < 0.01). Furthermore, all variables related to school bullying, whether in its traditional form or through technological means, showed a positive and statistically significant relationship with each other (r = 0.349, *p* < 0.01 [95% IC: 0.223, 0.469], for the lowest correlation between traditional victimization and technological perpetration; and r = 0.707, *p* < 0.01 [95% IC: 0.523, 0.817], for the highest correlation between technological victimization and perpetration). In addition, both experiencing and perpetrating peer violence via technological means were inversely associated with mental toughness (r = −0.172, *p* < 0.01 [95% IC: −0.270, −0.073] and r = −0.129, *p* < 0.05 [95% IC: −0.210, −0.045], respectively) and subjective vitality (r = −0.222, *p* < 0.01 [95% IC: −0.316, −0.119] and r = −0.205, *p* < 0.01 [95% IC: −0.305, −0.087], respectively). Finally, a direct relationship was observed between mental toughness and perceived subjective vitality (r = 0.496, *p* < 0.01 [95% IC: 0.404, 0.586]). Table 1 shows the Pearson correlation indices and statistical significance values for all index values and statistical significance of all pairs of variables considered.

### 3.2. Mediation Analysis

The PROCESS macro for SPSS [56] was used to examine both the direct and indirect effects, via mental toughness, of traditional and technological school bullying (from the perspectives of victims and aggressors) on students’ perceived subjective vitality. For each of the four mediation analyses (model 4), a bootstrapping procedure with 10,000 subsamples was performed. Gender and age were included as covariates in all analyses.

The results (Table 2, Table 3, Table 4 and Table 5) showed that, in relation to traditional bullying, levels of victimization were inversely related to subjective vitality, *c’* = −0.192, *p* = 0.030. In addition, greater attributions of mental toughness directly predicted higher feelings of vitality, *b* = 0.598, *p* < 0.001. However, mental toughness did not mediate the relationship between victimization and subjective vitality (see Table 2). On the other hand, the relationship between perpetrating aggressive acts against peers in face-to-face interaction and feelings of subjective vitality was fully mediated by perceived mental toughness, *a* × *b* = −0.148 [95% CI: −0.284, −0.020; *R*^2^ mediation effect size = 0.01, [0.00, 0.05]] (see Table 3).

When the analysis focused on examining aggressive acts, both suffered and perpetrated, through the use of technological tools, perceptions of victimization had a negative effect on participants’ subjective vitality, *c’* = −0.737, *p* = 0.001. Furthermore, this relationship was mediated by perceived mental toughness, *a* × *b* = (−0.578) × 0.574 = −0.331 [95% CI: −0.598, −0.123]. Being a victim of cyberbullying has a negative impact on mental toughness, which in turn contributes to an increase in perceived subjective vitality (see Table 4). In this context, two cases differing by one unit in experienced cybervictimization are estimated to differ by −0.331 units in subjective vitality through the effect of mental toughness [*R*^2^ mediation effect size = 0.03, [0.01, 0.07].

**Figure 1:**
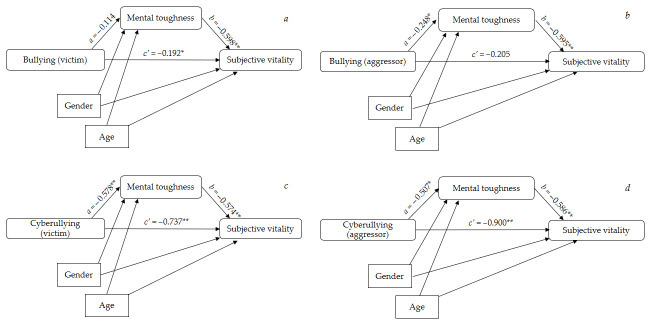
Graphical representations of the four mediational models (with two covariates included) explored. (**a**) IV bullying (victim); (**b**) IV (bullying aggressor); (**c**) IV (cyberbullying victim); (**d**) IV (cyberbullying aggressor). * *p* < 0.05; ** *p* < 0.01.

Similarly, perpetrating aggressive acts using electronic devices has a negative effect on perceived subjective vitality, *c’* = −0.900, *p* = 0.001. Additionally, mental toughness plays a statistically significant mediating role between these variables, *a* × *b* = (−0.507) × 0.586 = −0.297 [95% CI: −0.599, −0.080]. Thus, two cases that differ by one unit in perpetrated cybervictimization are estimated to differ by −0.297 units in subjective vitality through the influence of mental toughness [*R*^2^ mediation effect size = 0.02, [0.01, 0.05] (see Table 5). Figure 1 shows, by way of summary, the results obtained in the different mediation analyses.

## 4. Discussion

While previous research has demonstrated the detrimental effect of engaging in peer bullying behaviors on the psychological well-being of children and adolescents, fewer studies have focused on examining the variables or mechanisms that may mediate this relationship. To contribute to this line of evidence, the present study aimed to analyze the relationship between bullying and cyberbullying, from the perspective of both victims and aggressors, and subjective vitality in a sample of adolescents, considering the potential mediating effect of perceived mental toughness in this relationship. In addition, participants’ gender and age were considered as potential confounding variables. It was hypothesized that in situations of victimization, whether in its traditional form or through the use of technological tools, greater attribution of mental toughness might mitigate the likely negative relationship between experienced aggression and subjective vitality. From the aggressor’s perspective, although a negative relationship between participation in bullying behaviors and subjective vitality was predicted, the lack of prior evidence made us cautious about the specific role that mental toughness might play in this relationship.

The results partially confirmed the hypothesis, as high levels of mental toughness appeared to mitigate the negative effect of being a victim of peer bullying on subjective vitality. However, this was only observed when participants reported experiencing cybervictimization, not when victimization occurred during face-to-face interactions. One possible explanation may lie in the fact that during face-to-face interactions the victimized person is susceptible to physical aggression, an act that has no place when the behaviors aimed at causing harm to a peer occur through the use of technological means. In this sense, the threat and fear of physical pain together with the identification and public exposure to others would undermine the acquisition and strengthening of life control skills, emotional control and coping with challenges, characteristic of a high degree of mental toughness. Therefore, the absence of mental toughness or a reduced level of mental toughness would not be predictive of the victim’s subjective vitality [44,60]. Our results partially coincide with those of previous research in which the mediating variable used was resilience. In this regard, Gianesini and Brighi [34] found in a sample of Italian adolescents that high levels of resilience helped to mitigate the harmful effects associated with experiencing cyberaggression episodes, while also predicting higher scores on measures of psychosocial adjustment.

Similarly, Shemesh and Heiman [27] found that high positive self-concept combined with high levels of perceived resilience serves as significant mediators in the relationship between victimization and general psychological well-being. Their findings led to the conclusion that both self-concept and resilience act as protective mechanisms, seemingly buffering the decline in cognitive, emotional and social well-being associated with peer victimization. In the same vein, Narayanan and Belts [61] in their study of adolescents and young adults, confirmed that resilience significantly mediated the relationship between school bullying and perceived self-efficacy for coping with personal stressors and adversity. The results indicated that higher reported resilience was associated with greater perceived self-efficacy for managing the negative consequences of participating in bullying episodes [61].

Analyses conducted from the perspective of the perpetrator, whether through traditional means or technological tools, indicated a decrease in perceived life satisfaction as involvement in bullying behaviors toward peers increased. This finding is consistent with previous research suggesting that involvement in cyberbullying situations, regardless of whether one assumes the role of victim or perpetrator, is associated with the presence of depressive symptoms and a decrease in subjective well-being [28,62]. As noted by Mark et al. [13], the psychological health of aggressors may be severely compromised, as engaging in such acts appears to be associated with a lack of self-control, excessive impulsivity, adverse family circumstances, experienced hostility at home, and the need to achieve or regain a certain level of social status, aspects which may be indicative of poor self-esteem [63].

Additionally, our findings revealed the existence of a statistically significant mediating effect of mental toughness in the relationship between aggressive behavior and subjective vitality. In this context, the loss of energy and personal exhaustion associated with engaging in hostile acts towards peers appeared to be partially offset by the optimism, self-efficacy, and self-regulatory skills characteristic of mental toughness. As discussed above, engaging in cyberaggressive behavior has a negative impact on adolescents’ mental health. In this regard, Campbell et al. [64] concluded in their study of Australian adolescents that boys and girls who cyberbullied their peers had greater social difficulties and higher levels of stress, depression and anxiety than their non-aggressor peers. Similarly, Wong, Chan, and Cheng [65] suggested that engaging in aggressive behavior using technological devices is associated with the presence of negative emotions such as anger, sadness, frustration or fear.

This mediating effect of mental toughness in the relationship between bullying aggression and subjective vitality is consistent with the findings of Arslan and Allen [29]. In their study of Turkish adolescents, they found that positive psychological orientation mediated the relationship between bullying experiences (both victimization and perpetration) and subjective well-being. These findings are also consistent with those reported by Guerra and Bradshaw [66] and Kim, Furlong, and Dowdy [67], who concluded that positive psychological orientations had a statistically significant mediating effect on life satisfaction and emotional distress among adolescents. In this context, perceived self-efficacy, behavioral self-control, emotional regulation, empathy, and optimism, elements common to the construct of mental toughness, appear to play a prominent role in psychological well-being and in coping with emotional and behavioral problems in children and adolescents.

Thus, the attribution of high levels of mental toughness appears to help mitigate or reduce the personal distress derived from engaging in aggressive peer behaviors, particularly in situations where the aggressor may engage in such acts with the intention of restoring compromised self-esteem or social status. Aggressive acts between peers often have undesirable consequences; however, when such acts are accompanied by high levels of mental toughness, they may contribute to restoring some emotional balance for the perpetrator [38].

A variety of intervention programs have been proposed in an attempt to reduce bullying behaviors, as well as the negative psychological and emotional consequences derived from these actions. Some of these programs focus on modifying elements of the social climate within the school context with the aim of reducing these behaviors and fostering an atmosphere of safety and well-being both inside and outside the classroom [68]. Others, on the contrary, only contemplate the students involved in these situations. Whatever the nature of this intervention, a possible educational implication of our results points to the importance of the role that counselors, teachers and even parents can play in promoting certain personal qualities of students and children that seem to be associated with psychological well-being. As our results suggest, a high level of mental toughness restores in some sense the decline in subjective vitality associated with exposure to or commission of aggressive acts.

Suffering or experiencing episodes of bullying can lead to the presence of emotional (apathy, withdrawal), motivational (withdrawal, avoidance), and cognitive (hopelessness, fear) symptoms. These symptoms are incompatible with the attributes of control, defiance, commitment, and confidence that characterize mentally strong individuals [69]. In fact, mentally strong individuals would better manage the emotional distress caused by bullying situations because they perceive situations as more controllable, attribute greater capacity to themselves, increase their commitment to cope with adversity, and interpret problems as challenges rather than difficulties [70]. Thus, for the educational institution to encourage its students to acquire and strengthen a healthy resource such as increased mental toughness would help young people cope with the adversity and stress associated with bullying episodes. However, mental toughness is only one of many resources of a resilient nature, and even individuals with a high degree of mental toughness have limitations that make coping with stress a difficult task [71]. Nevertheless, promoting mental toughness can be an ingredient in resilient coping programs. In this sense, previous studies suggest that promoting positive psychological characteristics such as resilience, self-concept and even the feeling of belonging to the school institution [27,30,33] reduces the loss of psychological well-being among those involved in these scenarios.

Despite these findings, the study is not without limitations. First, the information was collected through self-report questionnaires, which leaves open the possibility that participants’ responses may have been influenced by factors such as social desirability, unconventional reporting tendencies, or even memory distortions. Efforts were made to minimize the latter by limiting participants’ judgements of bullying behavior, both experienced and perpetrated, to the previous two months. Secondly, the study has cross-sectional design, which prevents the establishment of causal relationships between the variables of interest. Therefore, future studies are encouraged to use both longitudinal and experimental designs to replicate or extend the results obtained here. Third, although bullying and cyberbullying behaviors were examined from the perspectives of victims and aggressors, the possibility that many participants may simultaneously occupy both roles, victim and aggressor, was not addressed. Previous studies have considered this dual category because of the significant relationship that the victim–perpetrator condition has with the psychological health and well-being of children and adolescents. Finally, the fact that the sampling in this study was carried out by convenience may limit the generalizability of the results obtained.

## 5. Conclusions

The results of the present study suggest that perceived mental toughness was found to act as a psychological variable that helps to buffer the decline in subjective vitality among adolescents involved in virtual bullying scenarios, whether as victims or perpetrators. However, this mediating effect was not observed when bullying occurred through face-to-face interactions. Nevertheless, it is recommended that educational institutions promote activities aimed at fostering positive psychological characteristics to compensate for the loss of well-being experienced by individuals involved in bullying situations.

## Figures and Tables

**Table 1:** Mean values, standard deviations, and Pearson correlation indexes among the different study variables.

	Mean (SD)	1	2	3	4	5	6	7
Age	13.29 (1.66)	1						
Bullying (victim)	1.79 (0.80)	−0.243 **	1					
Bullying (aggressor)	1.44 (0.55)	−0.031	0.493 **	1				
Cyberbullying (victim)	1.18 (0.28)	0.007	0.459 **	0.473 **	1			
Cyberbullying (aggressor)	1.11 (0.23)	0.062	0.349 **	0.594 **	0.707 **	1		
Mental toughness	5.43 (0.98)	−0.128 *	−0.066	−0.130*	−0.172 **	−0.129 *	1	
Subjective vitality	4.83 (1.30)	−0.169 **	−0.112 *	−0.108	−0.222 **	−0.205 **	0.496	1

* *p* < 0.05; ** *p* < 0.01; SD = standard deviation.

**Table 2:** Coefficient value and statistical significance of the regression analysis between bullying (victimization) and subjective vitality, using as mediator the score obtained in perceived mental toughness.

				Consequent							
				*M* (Mental Toughness)					*Y* (Subjective Vitality)		
Antecedent				Coeff.	SE	*p*			Coeff.	SE	*p*
*X* Bullying (Victimization)			*a*	−0.114	0.086	0.186		*c’*	−0.192	0.067	0.030
*M* (Mental toughness)								*b*	0.598	0.067	<0.001
*C*_1_ (Gender)			*f*_1_	−0.207	0.112	0.065		*g*_1_	−0.216	0.129	0.095
*C*_2_ (Age)			*f*_2_	−0.095	0.036	0.008		*g*_2_	−0.113	0.037	0.002
Constant			*I*_1_	7.222	0.559	<0.001		*i*_2_	3.770	0.799	<0.001
											
				*R*^2^ = 0.038					*R*^2^ = 0.280		
				*F*(3, 308) = 3.610, *p* = 0.014					*F*(3, 307) = 33.684, *p* < 0.001		

**Table 3:** Coefficient value and statistical significance of the regression analysis between bullying (aggressor) and subjective vitality, using as mediator the score obtained in perceived mental toughness.

				Consequent							
				*M* (Mental Toughness)					*Y* (Subjective Vitality)		
Antecedent				Coeff.	SE	*p*			Coeff.	SE	*p*
*X* Bullying (Aggression)			*a*	−0.248	0.123	0.045		*c’*	−0.205	0.120	0.088
*M* (Mental toughness)								*b*	0.595	0.067	<0.001
*C*_1_ (Gender)			*f*_1_	−0.231	0.111	0.038		*g*_1_	−0.252	0.130	0.053
*C*_2_ (Age)			*f*_2_	−0.086	0.034	0.012		*g*_2_	−0.094	0.037	0.012
Constant			*I*_1_	7.284	0.514	<0.001		*i*_2_	3.526	0.797	<0.001
											
				*R*^2^ = 0.049					*R*^2^ = 0.274		
				*F*(3, 308) = 4.827, *p* = 0.003					*F*(4, 307) = 28.813, *p* < 0.001		

**Table 4:** Coefficient value and statistical significance of the regression analysis between cyberbullying (victimization) and subjective vitality, using as mediator the score obtained in perceived mental toughness.

				Consequent							
				*M* (Mental Toughness)					*Y* (Subjective Vitality)		
Antecedent				Coeff.	SE	*p*			Coeff.	SE	*p*
*X* Cyberbullying (Victimization)			*a*	−0.578	0.188	0.002		*c’*	−0.737	0.215	0.001
*M* (Mental toughness)								*b*	0.574	0.064	<0.001
*C*_1_ (Gender)			*f*_1_	−0.219	0.109	0.046		*g*_1_	−0.243	0.124	0.051
*C*_2_ (Age)			*f*_2_	−0.086	0.035	0.014		*g*_2_	−0.094	0.039	0.018
Constant			*I*_1_	7.592	0.551	<0.001		*i*_2_	4.210	0.789	<0.001
											
				*R*^2^ = 0.059					*R*^2^ = 0.293		
				*F*(3, 308) = 6.395, *p* < 0.001					*F*(3, 307) = 31.769, *p* < 0.001		

**Table 5:** Coefficient value and statistical significance of the regression analysis between cyberbullying (aggressor) and subjective vitality, using as mediator the score obtained in perceived mental toughness.

				Consequent							
				*M* (Mental Toughness)					*Y* (Subjective Vitality)		
Antecedent				Coeff.	SE	*p*			Coeff.	SE	*p*
*X* Cyberbullying (Aggressor)			*a*	−0.507	0.235	0.032		*c’*	−0.900	0.265	0.001
*M* (Mental toughness)								*b*	0.586	0.064	<0.001
*C*_1_ (Gender)			*f*_1_	−0.221	0.110	0.045		*g*_1_	−0.240	0.124	0.053
*C*_2_ (Age)			*f*_2_	−0.082	0.035	0.020		*g*_2_	−0.086	0.039	0.030
Constant			*I*_1_	7.421	0.560	<0.001		*i*_2_	4.163	0.784	<0.001
											
				*R*^2^ = 0.044					*R*^2^ = 0.292		
				*F*(3, 308) = 4.746, *p* = 0.003					*F*(3, 307) = 31.704, *p* < 0.001		

## Data Availability

The data presented in this study are available on request from the corresponding author due to privacy, legal, and ethical reasons of this project.
